# Electrical stimulation facilitates NADPH production in pentose phosphate pathway and exerts an anti-inflammatory effect in macrophages

**DOI:** 10.1038/s41598-023-44886-x

**Published:** 2023-10-19

**Authors:** Mikiko Uemura, Noriaki Maeshige, Atomu Yamaguchi, Xiaoqi Ma, Mami Matsuda, Yuya Nishimura, Tomohisa Hasunuma, Taketo Inoue, Jiawei Yan, Ji Wang, Hiroyo Kondo, Hidemi Fujino

**Affiliations:** 1 Department of Rehabilitation Science, Kobe University Graduate School of Health Sciences, 7-10-2 Tomogaoka, Kobe, Hyogo 654-0142 Japan; 2 Department of Rehabilitation, Faculty of Health Sciences, Kansai University of Welfare Sciences, Kashiwara, Japan; 3 Graduate School of Science, Technology and Innovation, Kobe University, Kobe, Japan; 4 Engineering Biology Research Center, Kobe University, Kobe, Japan; 5 Department of Emergency, Disaster and Critical Care Medicine, Hyogo Medical University, Nishinomiya, Japan; 6 School of Life Sciences and Technology, ShanghaiTech University, Shanghai, China; 7 Department of Toxicology and Sanitary Chemistry, School of Public Health, Capital Medical University, Beijing, China; 8 Department of Food Science and Nutrition, Nagoya Women’s University, Nagoya, Japan

**Keywords:** Immunology, Health care

## Abstract

Macrophages play an important role as effector cells in innate immune system. Meanwhile, macrophages activated in a pro-inflammatory direction alter intracellular metabolism and damage intact tissues by increasing reactive oxygen species (ROS). Electrical stimulation (ES), a predominant physical agent to control metabolism in cells and tissues, has been reported to exert anti-inflammatory effect on immune cells. However, the mechanism underlying the anti-inflammatory effects by ES is unknown. This study aimed to investigate the effect of ES on metabolism in glycolytic-tricarboxylic acid cycle (TCA) cycle and inflammatory responses in macrophages. ES was performed on bone marrow-derived macrophages and followed by a stimulation with LPS. The inflammatory cytokine expression levels were analyzed by real-time polymerase chain reaction and ELISA. ROS production was analyzed by CellRox Green Reagent and metabolites by capillary electrophoresis-mass spectrometry. As a result, ES significantly reduced proinflammatory cytokine expression levels and ROS generation compared to the LPS group and increased glucose-1-phosphate, a metabolite of glycogen. ES also increased intermediate metabolites of the pentose phosphate pathway (PPP); ribulose-5-phosphate, rebose-5 phosphate, and nicotinamide adenine dinucleotide phosphate, a key factor of cellular antioxidation systems, as well as α-Ketoglutarate, an anti-oxidative metabolite in the TCA cycle. Our findings imply that ES enhanced NADPH production with enhancement of PPP, and also decreased oxidative stress and inflammatory responses in macrophages.

## Introduction

Macrophages are effector cells of the innate immune system that play an essential role in the defense against pathogenic infections. They phagocytose pathogens and produce inflammatory cytokines such as interleukin (IL)-6 or tumor necrosis factor (TNF)-α, which can induce activation, proliferation, and chemotaxis of neighboring cells and macrophages^1^. Inflammatory-stimulated macrophages also produce reactive oxygen species (ROS) for pathogen challenge^2^. Although reactive oxygen is necessary as a biophylaxis reaction, its overproduction activates nuclear factor-kappa B (NF-kB)/mitogen-activated protein kinase, leading to the secretion of pro-inflammatory cytokines^3,4^. Moreover, overexpression of inflammatory cytokines could potentially trigger systemic inflammatory response syndrome and cause cell damage^5^. Thus, it is crucial to control inflammatory response and oxidative stress in macrophages to prevent tissue and organ injuries. Macrophage metabolism is closely related with inflammation. Glucose is converted to glucose-6-phosphate (G6P) to enter the glycolysis or the pentose phosphate pathway (PPP), and fluctose-6-phosphate (F6P) is produced when inflammation is occurred in macrophages^6^. The PPP is a branch of glycolytic metabolism that produces nicotinamide adenine dinucleotide phosphate (NADPH) via G6P. NADPH, primarily produced by PPP, is essential for cell survival under oxidative stress and inflammation^6,7^. Pyruvate, the final metabolite of glycolytic pathway, is converted to lactate or acetyl-CoA, and the tricarboxylic acid (TCA) cycle is initiated from acetyl-CoA. The TCA cycle is broken and citrate and succinate are accumulated in macrophages under inflammatory stimuli. In the TCA cycle, citrate is converted to cis-Aconitate, and itaconate produced from cis-Aconitate regulates the inflammatory cascade with activation of NRF-2 which has anti-inflammatory effect in macrophages^8,9^, while succinate enhances inflammatory response and production of inflammatory cytokines^10^.

Electrical stimulation (ES) and ultrasound therapy are used as non-invasive treatments mainly for chronic inflammation-based diseases such as peritendinitis, arthritis and pressure injury, and their therapeutic effects have been documented^11–13^. The anti-inflammatory effect of ultrasound has been reported to involve an increase in itaconate production in macrophages^14^. However, studies on the effects of ES on immune cells are limited. A few studies reported that ES decreased pro-inflammatory cytokine expressions in lipopolysaccharide (LPS) stimulated immune cells and injury tissues^15–17^. Following these reports, ES has been used as an anti-inflammatory regimen; however, the underlying mechanism and effect on macrophage metabolism, a critical target of inflammatory regulation, have not been explored yet. This study investigated the metabolism in glycolytic-TCA cycle and the anti-inflammatory/anti-oxidative effects in ES-treated macrophages.

## Results

### ES has no cytotoxic effects on bone marrow-derived macrophages (BMDMs)

**Figure 1:**
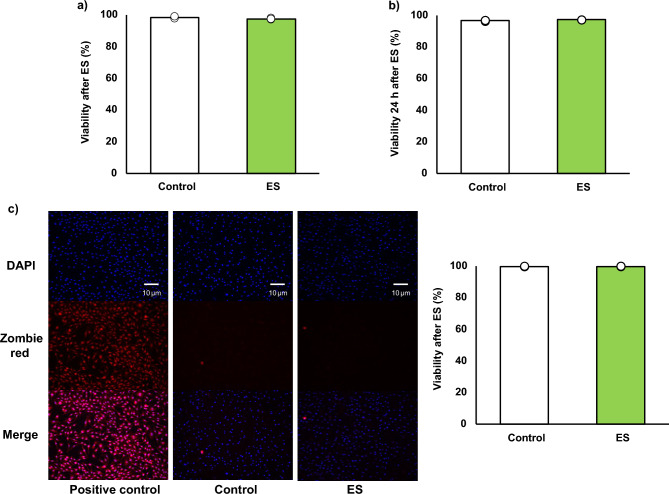
Cell viability. Viability was calculated as a ratio of dead cells to whole cell. (**a**, **c**) ES did not have cytotoxicity on BMDMs after ES. Figure 1a shows viability immediately after ES. Figure 1c shows the zombie red staining and ES did not increase dead cells, and Fig. 1c shows the ratio of zombie red stained cells to DAPI stained cells. Blue; nucleus, and red; dead cell. (**b**) The viability was maintained 24 h after ES as well as that of the control group. The statistical differences between the control and ES groups were tested by Student’s-t test. n = 3 per group. Data are presented as mean ± SD.

Figure 1a, b show the cell viabilities of BMDMs after ES calculated by trypan blue staining. Stained cells (dead cells) and living cells were counted and viability, a ratio of the stained cells to whole cell, was calculated. Cell viability immediately after electrical stimulation was 98.42 ± 0.46% for the control group, and 97.69 ± 0.47% for the ES group (Fig. 1a), and it was maintained 24 h after ES (Fig. 1b, 96.54 ± 0.31%, and 97.14 ± 0.06%, respectively). Figure 1c shows the result of DAPI (nucleus) and zombie red staining (dead cells). In the ES group, as in the control group, few dead cells were stained, and viability was not decreased (99.52% ± 0.12%, for the control group, and 99.69 ± 0.08% for the ES group). ES did not show cytotoxicity in BMDMs compared to the control and the cell viability was maintained 24 h after ES.

### ES suppressed inflammatory response and oxidative stress in BMDMs

**Figure 2:**
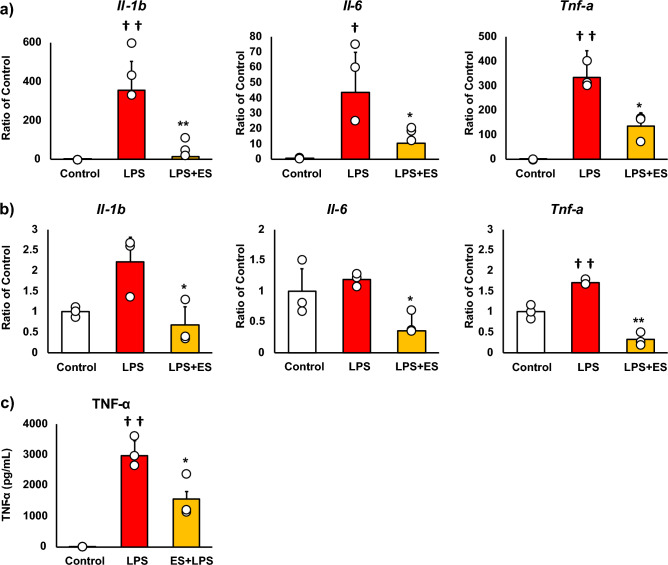
Expression of pro-inflammatory cytokines. (**a**) LPS was added to BMDMs for 1.5 h after ES, and *Il-1b*, *Il-6*, and *Tnf-a* mRNA expression was analyzed. These results represent the ratios in the control group. LPS stimulation significantly increased *Il-1b*, *Il-6,* and *Tnf-a* mRNA expressions. ES suppressed *Il-1b*, *Il-6*, and *Tnf-a* expression in response to LPS stimulation (*Il-1β*, *p* < 0.01; *Il-6*, and *Tnf-a*, *p* < 0.05). (**b**) ES also suppressed *Il-1b*, *Il-6*, and *Tnf-α* mRNA expressions 24 h after LPS stimulation compared to the LPS group (*Il-1β* and *Il-6*, *p* < 0.05; *Tnf-a*, *p* < 0.01). (**c**) TNF-α protein level was significantly suppressed 24 h after LPS stimulation (*p* < 0.05). Statistical differences between the control and ES groups were tested using Turkey–Kramer test. n = 3 per group. †*p* < 0.05, versus control group, ††*p* < 0.01, versus control group, *p < 0.05, versus LPS group, and **p < 0.01, versus LPS group. Data are presented as mean ± SD.

**Figure 3:**
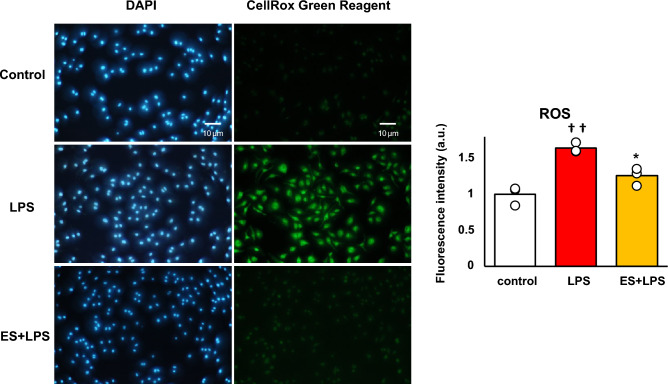
ROS generation. BMDMs were stained with DAPI or CellRox Green Reagent. CellRox Green Reagent is a DNA dye and binds to DNA when oxidation. LPS-stimulus BMDMs were stained (middle) compared to the control group (higher) and the ES + LPS group (lower). The fluorescence intensities of each group were calculated by Image J and compared to the control group. The intensity of the LPS group was significantly higher than that of the control group (*p* < 0.01), but the ES + LPS group suppressed that intensity compared to LPS group (*p* < 0.05). Blue; DAPI, and green; ROS. The statistical differences between these groups were tested by Tukey–Kramer test. n = 3 per group. *p < 0.05, versus LPS group, and ††*p* < 0.01, versus control group. Data are presented as mean ± SD.

Figure 2 shows the mRNA expressions of pro-inflammatory factors, and these results are presented as relative values with the LPS non-stimulated control group. LPS significantly increased *Il-1b, Il-6, and Tnf-a* mRNA expressions against the control group. Whereas, ES significantly suppressed LPS-induced upregulation of *Il-1b*, *Il-6*, and *Tnf-a* mRNA expressions (*Il-1b*; *p* < 0.01, *Il-6*; *p* < 0.05, and *Tnf-a*; *p* < 0.05, vs. LPS group, Fig. 2a), and these inflammatory cytokine expressions were still suppressed 24 h after LPS treatment in the ES + LPS group (*Il-1b*; *p* < 0.05, *Il-6*; *p* < 0.05, *TNF-a*; *p* < 0.01, vs. LPS group, Fig. 2b). Regarding TNF-α expression, LPS significantly promoted TNF-α protein level 24 h after LPS treatment (*p* < 0.01, vs. control group). On the other hand, TNF-α protein level was also significantly suppressed in the ES + LPS group (*p* < 0.05, vs. LPS group, Fig. 2c). Figure 3 shows ROS staining. LPS significantly increased ROS production compared to the control (Fig. 3, *p* < 0.01), whereas ES suppressed ROS production induced by LPS (*p* < 0.05, vs. LPS).

### ES facilitates the pentose phosphate pathway and NADPH production

**Figure 4:**
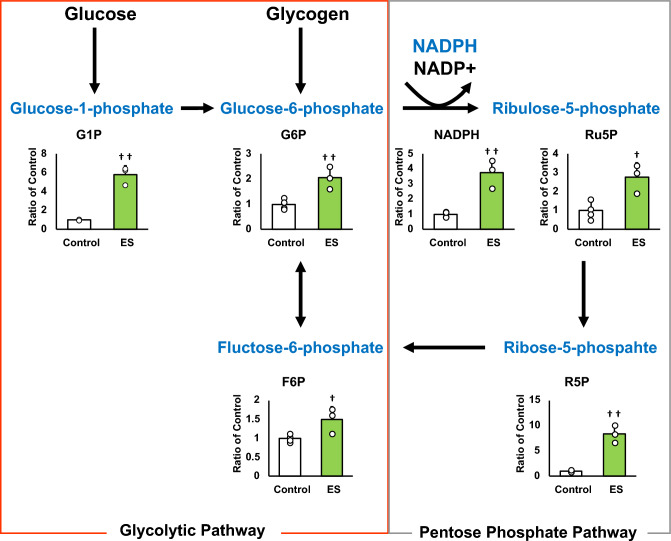
Metabolites and glycolytic metabolic pathway. All metabolite productions were measured by CE/MS, and these results present the rations of the control group. ES increased G1P, G6P, and F6P productions, the composition of glycogen and glucose (G1P and G6P, *p* < 0.01, F6P, *p* < 0.05). ES also increased Ru6P, R5P and NADPH productoins which were produced in PPP (Ru5P, *p* < 0.05, R5P and NADPH, *p* < 0.01). The data were compared to those of the control group. Student’s t-test. n = 4 for control group, and n = 3 for ES group. †*p* < 0.05, versus control group, and ††*p* < 0.01 versus control group. Data are presented as mean ± SD.

**Figure 5:**
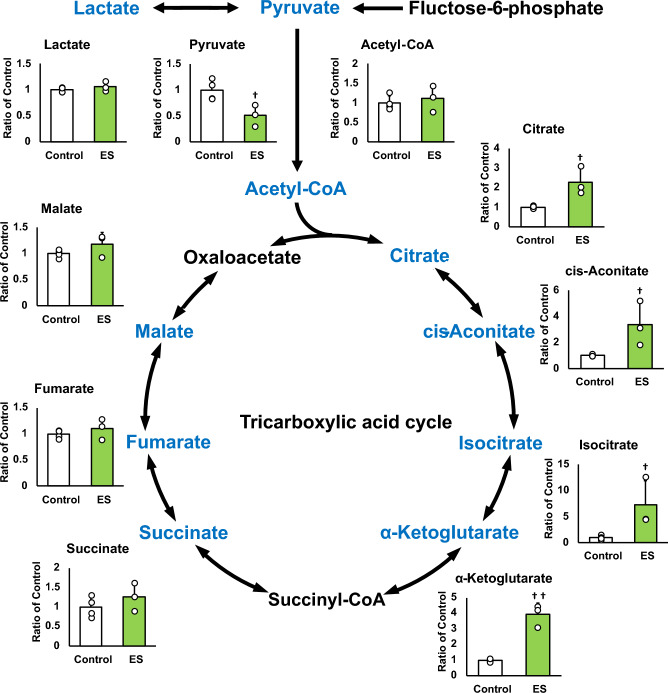
Metabolites in TCA cycle. All metabolite productions were measured by CE/MS, and these results present the rations of the control group. ES decreased pyruvate production but that of citrate, cis-Aconitate, isocitrate and AKG productions were increased by ES, however, that of succinate, fumarate, and malate puroductions were not increased. Statistical differences between the control and ES groups were tested using Student’s t-test. n = 4 for control group, and n = 3 for ES group. †*p* < 0.05, versus control group Data are presented as mean ± SD.

**Figure 6:**
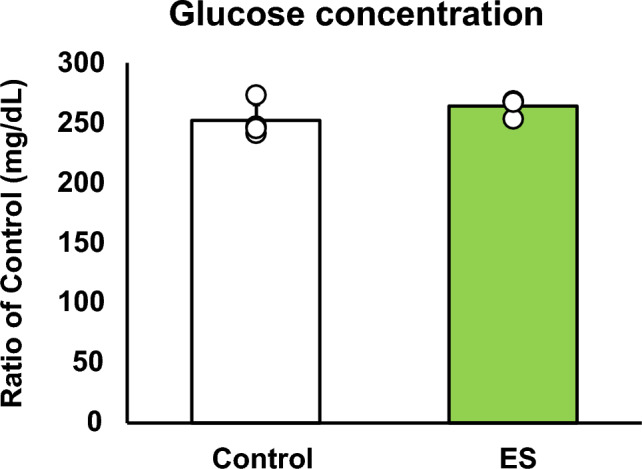
Glucose concentration in the culture medium. ES did not affect the glucose concentration in the culture medium compared to that in the control group. Statistical differences between the control and ES groups were tested using Student’s t-test. n = 3 per group. Data are presented as mean ± SD.

Figures 4 and 5 show the intracellular metabolites levels, and these results are presented as relative values with the control group. The abundance of metabolites in macrophages following ES was analyzed to investigate whether ES produced metabolites with anti-inflammatory or antioxidant effects. In glycolytic pathway, ES significantly increased the productions of D-glucose-1-phosphate (G1P), which is a metabolite of glycogen, G6P, and F6P compared to the control group (G1P and G6P; *p* < 0.01, F6P; *p* < 0.05 vs. control group, Fig. 4). There are two pathways of metabolism from G6P to F6P; glycolytic pathway and PPP. ES also increased ribulose-5-phosphate (Ru5P), ribose-5-phosphate (R5P), and NADPH productions which are intermediate metabolites of PPP (R5P, and NADPH; *p* < 0.01, and Ru5P; *p* < 0.05, vs. control group, Fig. 4). Thus, these results show that ES enhanced glucose and glycogen metabolism. In glycolytic metabolism, F6P is metabolized to pyruvate and pyruvate is transferred to lactate or the TCA cycle via Acetyl-CoA. ES increased F6P production but decreased pyruvate production (*p* < 0.05 vs. control group, Fig. 5), and there was no difference in lactate production or acetyl-CoA production between the ES and control groups. Regarding metabolites of the TCA cycle, citrate, cis-Aconitate, isocitrate, and α-Ketoglutarate (AKG) productions were significantly increased by ES (Citrate, cis-Aconitate, isocitrate; *p* < 0.05, vs. control group, AKG; *p* < 0.01, vs. control group, Fig. 5). However, succinate, which is converted from AKG via Succinyl-CoA, fumarate, and malate productions were not increased by ES (Fig. 5). The level of itaconate, converted from cis-Aconitate, was not altered by ES (see Supplementary file S1). Next, the glucose concentration in the culture medium was measured to examine whether the increased G1P production by ES was due to increased intracellular metabolism or increased glucose uptake in the culture medium, and no significant difference was observed between the control and ES groups (Fig. 6).

## Discussion

This study is the first report to show that ES exerts anti-inflammatory and antioxidant effects on macrophages and also alters intracellular metabolism of macrophages to PPP. The present study showed that inflammatory cytokine expressions and ROS production were suppressed by ES on BMDMs prior to LPS stimulation. In the present study, ES on BMDMs increased the production of G1P, a metabolite of glucose, approximately 4.5 times, and G6P, which is converted from glycogen, while ES did not affect the glucose uptake of culture medium. These results suggest that the increase in G1P production by ES is due to intracellular glucose metabolism, not increased glucose uptake. In addition, ES increased NADPH, Ru5P, and R5P which are involved in PPP. NADPH is generated when G6P is converted to Ru5P, and Ru5P is finally converted to F6P via R5P in PPP^18^. Hence, our results suggested that ES enhanced glycolytic metabolism via glycogenolysis, leading to PPP activation in BMDMs.

Immune cell functions and PPP are closely related. CD8 + memory T cells produce G6P by glycolytic metabolism and linking it to PPP, which induces anti-oxidant effects^19^. NADPH produced via PPP mediates the reaction in glutathione from oxidized to reduced status, which recovers anti-oxidative capacity in cells^9,20^. In the present study, ES suppressed ROS generation of BMDMs against LPS stimulation and increased NADPH production. Thus, ROS-reducing effect of ES might have resulted from the increase of antioxidant capacity of BMDMs due to enhancement of NADPH production in PPP. Meanwhile, pyruvate, which is converted from F6P and expended for the TCA cycle, was decreased by ES. It is reported the reaction between F6P and G6P is reversible^21^. Therefore, ES on BMDMs might enhance PPP with the reduced conversion of F6P to pyruvate, converting F6P to G6P, forming a pentose phosphate circuit, resulted in NADPH accumulation. Regarding to the TCA cycle, citrate, cis-Aconitate, isocitrate, and AKG were increased by ES, but succinate, converted from AKG was not increased. AKG has anti-oxidative effects with regulation of the constitutive-androstane-receptor^22^. Therefore, increased AKG could also contribute the anti-oxidative effect by ES. As for the immunometabolism in pathological conditions, glycolytic macrophages stimulated with LPS interrupted the TCA cycle, where conversion from isocitrate into AKG is blocked, leading to accumulation of succinate. In this study, ES increased G1P and enhanced PPP but not succinate accumulation with an increased AKG level. These results support the specificity of ES in the metabolic change of macrophages and suggest its therapeutic potential. In the inflammatory phase of rheumatoid arthritis, an inflammatory disease, most of the macrophages in the peripheral blood and synovial tissue are M1 macrophages, which cause tissue damage through increased ROS production, NF-kB activation, and excessive production of inflammatory cytokines such as IL-6^23^. Many studies and reviews have revealed the relationship between oxidative stress and inflammatory response^2,24^. ROS introduced by LPS activates the production of inflammatory cytokines such as IL-6, IL-1β, and TNF-α^3^. Furthermore, suppression of ROS generation inhibits caspase1 activation^25^, resulting in the cleavage of pro-IL-1β and IL-18. Consequently, the activated forms of pro-IL-1β and IL-18 induce an inflammatory response, demonstrating the link between caspase1 activation and the promotion of inflammation^26,27^. The reduction of ROS generation by ES suppressed caspase1, leading to suppression of IL-1β expression in inflammatory macrophages^28^. Thus, it is suggested that ES provides inhibitory effect of pro-inflammatory cytokine expressions in inflammatory BMDMs by protecting against oxidative stress. Regarding the effects of other physical agents, ultrasound showed anti-inflammatory effects via increased itaconate concentration in macrophages^14^; however, ES did not alter the itaconate level in BMDMs (see Supplementary file S1). This result also showed the unique effect of electrical stimulation different from ultrasound. The limitation of this study is that the mechanisms by which ES enhances glycogenolysis and branches to PPP have not been elucidated, and the causal relationship between changes in intracellular metabolites following ES and inflammatory cytokine expressions is not explored well.

In conclusion, ES might suppress oxidative stress and inflammatory responses in BMDMs with enhancement of NADPH generation in PPP. Our findings show that ES could be a therapeutic and preventative treatment against aberrant inflammatory conditions.

## Material and methods

### Cell culture

BMDMs were obtained by harvesting bone marrow from 7-week-old male C57BL/6NCrSlc mice and cultured in RPMI 1640 medium (186–02155, Fujifilm Wako, Osaka, Japan) supplemented with 10% fetal bovine serum (FBS), 1% penicillin/streptomycin, 1% L-glutamine, and 25% L929 cell supernatant for 8 days. BMDMs were plated in 35-mm tissue culture dishes for the experiments. BMDMs were treated with 100 ng/mL LPS for 1.5 h or 24 h in the LPS and the ES + LPS groups. This study was approved by the Institutional Animal Care and Use Committee and performed according to the Kobe University Animal Experimentation Regulations (P210803).

### Electrical stimulation

**Figure 7:**
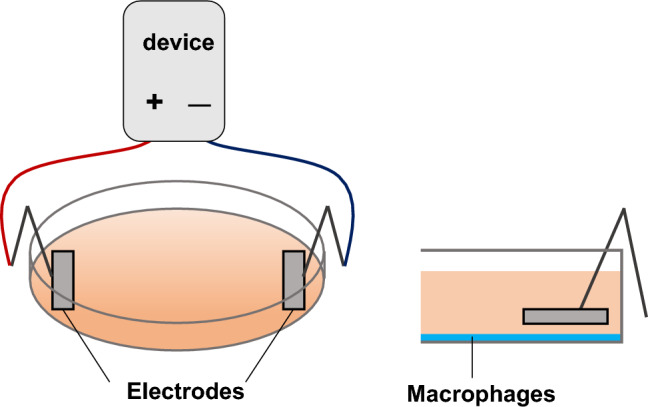
Electrical stimulation. BMDMs were electrical stimulated with platinum electrodes.

In this study, ES (intensity: 200 μA, frequency: 2 Hz, and duration: 250 ms) was adopted because it was reported to activate cell migration and proliferation for in vitro studies,^29,30^ and it is reported that ES with these parameters promoted healing of pressure injuries, a disease with chronic inflammation, in clinical study^31^. Culture media were replaced with FBS-free RPMI 1640, and ES was conducted with platinum electrodes for 4 h at 37 °C in a 5% CO_2_ incubator in the ES and the ES + LPS groups (Fig. 7). ES was conducted before LPS treatment in the ES + LPS group.

### Cell viability

Cell viability after ES was measured to assess the cytotoxicity of ES treatment. BMDMs after 4-h-ES were washed with phosphate buffered saline (PBS) and stained with Trypan Blue Solution (Thermo Fisher, MA, USA) for trypan blue staining. Similarly, dishes were washed with PBS and incubated for 15 min with Zombie Red™ Fiable Viability Kit (1:1000, BioLegend, CA, USA). The dishes were washed with PBS and incubated for 30 min at room temperature with 4% formaldehyde. DAPI (1:1,000; Dojindo, Kumamoto, Japan) was added, and the cells were incubated for 5 min at 37 °C after washing with PBS. The stained cells were observed with a fluorescence microscope (BZ-X800, Keyence, Osaka, Japan). The number of living and dead cells was counted and cell viability was calculated.

### Real time-polymerase chain reaction (RT-PCR)

mRNA was isolated using TRIzol reagent (Thermo Fisher), and reverse transcription was performed using the iScript cDNA Synthesis Kit (Bio-Rad, CA, USA). RT-PCR was performed using targeted gene primers with the following cycling parameters: 95 °C (3 min), 40 cycles of 95 °C (10 s), and 60 °C (30 s) using SYBR Green (Invitrogen, MA, USA) with the StepOne™ Real-Time PCR System (Thermo Fisher). mRNA expression levels of *Il-1b*, *Il-6*, *Tnf-a*, and *hypoxanthine phosphoribosyltransferase (Hprt)* were measured. The primer sequences are shown in Supplementary file S2. Relative fold changes in expression were calculated by normalizing to *Hprt*, and the data were analyzed using the 2^−ΔΔCT^ method. The results are shown as values relative to the control group.

### Enzyme-linked immunosorbent assay (ELISA)

ELISA was performed to analyze the level of TNF-α in the cell supernatant. TNF-α was detected with Mouse TNF-α DuoSet ELISA (R&D systems, MN, USA, DY410-05) according to the manufacturer’s protocol. 

### Detection of ROS

ROS production was analyzed using the CellRox Green Reagent (Invitrogen). The reagent was added to each dish at a concentration of 10 μmol/L, and the cells were incubated for 30 min at 37 °C in a 5% CO_2_ incubator. The dishes were washed with PBS and incubated for 15 min at room temperature with 3.7% formaldehyde. DAPI (1:1,000; Dojindo) was added, and the cells were incubated for 5 min at 37 °C after washing with PBS. The stained cells were observed with a fluorescence microscope (Olympus, Tokyo, Japan). The fluorescence intensity of each dish was analyzed using Image J (Version 1.53u, NIH, MD, USA) in according to Shihan’s study^32^, and the results were analyzed as a ratio to the control group.

### Metabolite analysis

Eighty percent MeOH [water containing 50 μM ( +)-10-camphorsulfonic acid, 400 μM L-methionine sulfone, and 400 μM piperazine-1,4-bis (2-ethanesulfonic acid)] was added to each dish and incubated for 15 min at − 80 °C. Cells were scraped and centrifuged at 14,000 × g for 5 min, and the supernatant was centrifuged at 14,000 × g for 90 min at 4 °C using a 5 kDa cut-off membrane (Merck Millipore, MA, USA) to remove the solubilized protein. The dried metabolites concentrated by evaporation of the aqueous layer extracts with a FreeZone 2.5 Plus freeze-dry system (Labconco, Kansas City, MO) were dissolved in Milli-Q water. The intracellular metabolites were analyzed using a capillary electrophoresis-mass spectrometry (CE/MS, Agilent G7100; MS, Agilent G6224AA LC/MSD TOF; Agilent Technologies, Palo Alto, CA) controlled by the MassHunter Workstation Data Acquisition software (Agilent Technologies)^33^.

### Glucose measurement

One milliliter of culture medium from each dish was collected and centrifuged at 1500 × g for 5 min. Then, 5 μL of the medium and 750 μL of the color reagent (Wako) were mixed, and the absorbance was recorded at 505 nm.

### Statistical analysis

Statistical analysis was conducted with Easy R (EZR; Saitama Medical Center, Jichi Medical University, Saitama, Japan), a graphical user interface for R (The R Foundation for Statistical Computing, Vienna, Austria)^34^. Student’s t-test was followed by Shapiro–Wilk test and F-test and one-way analysis of variance (ANOVA) test was followed by Turkey's multiple comparisons as a post-hoc analysis.

### Ethics approval and informed consent

This study was approved by the Institutional Animal Care and Use Committee and performed according to the Kobe University Animal Experimentation Regulations (P210803). This study was performed in accordance with ARRIVE guidelines.

### Supplementary Information

Supplementary Legends.

Supplementary Figure S1.

Supplementary Table S2.

## Data Availability

The authors confirm that the data supporting the findings of this study are available within the article.

## Abbreviations

LPS: Lipopolysaccharide
ROS: Reactive oxygen species
ES: Electrical stimulation
BMDMs: Bone marrow-derived macrophages
NADPH: Nicotinamide adenine dinucleotide phosphate
G1P: Glucose-1-phosphate
G6P: Glucose-6-phosphate
F6P: Fluctose-1-phosphate
Ru5P: Ribulose-5-phosphate
R5P: Ribose-5-phosphate
AKG: α-Ketoglutarate
