# Supplementary material for: Electrical stimulation facilitates NADPH production in pentose phosphate pathway and exerts an anti-inflammatory effect in macrophages
Source: Sci Rep. 2023 Oct 19;13:17819. doi: 10.1038/s41598-023-44886-x (PMC10587116; doi:10.1038/s41598-023-44886-x)
Supplement: Supplementary file 1 — Supplementary Legends. [file 41598_2023_44886_MOESM1_ESM.docx]

Supplementary file S1 Itaconate production. Itaconate production was measured by CE/MS. ES did not increase itaconate level. Statistical differences between the control and ES groups were tested using Student’s t-test. n = 3 per group. Data are presented as mean ± SD.
