# Supplementary material for: Electrical stimulation facilitates NADPH production in pentose phosphate pathway and exerts an anti-inflammatory effect in macrophages
Source: Sci Rep. 2023 Oct 19;13:17819. doi: 10.1038/s41598-023-44886-x (PMC10587116; doi:10.1038/s41598-023-44886-x)
Supplement: Supplementary file 3 — Supplementary Table S2. [file 41598_2023_44886_MOESM3_ESM.docx]

Supplemental file S2. Primers sequences

| Primer | Sequences |
| --- | --- |
| *Hprt* – Forward | 5’-GCTTGCTGGTGAAAAGGACCTCTCGAAG-3’ |
| *Hprt* – Reverse | 5'-CCCTGAAGTACTCATTATAGTCAAGGGCAT-3’ |
| *Il-1β* – Forward | 5’-ACAAAGCCAGAGTCCTTCAGAGAG-3’ |
| *Il-1β* – Reverse | 5’-TTGGATGGTCTTGGTCCTTAGCCA-3’ |
| *Il-6* – Forward | 5’-CCTATGTCTCAGCCTCTTCT-3’ |
| *Il-6* - Reverse | 5’-GGGAACTTCTCATCCCTTTG-3’ |
| *Tnf-α* – Forward | 5’-CAGCATAGAGCAGGACATGGAG-3’ |
| *Tnf-α* - Reverse | 5’-GAACAGCGGTAGTATCAGCCAG-3’ |
